# A virtual reality paradigm to assess episodic memory: Validation-dataset for six parallel versions and a structured behavioral assessment

**DOI:** 10.1016/j.dib.2020.105279

**Published:** 2020-02-13

**Authors:** Yvonne Höller, Christopher Höhn, Fabian Schwimmbeck, Gaën Plancher, Eugen Trinka

**Affiliations:** a Faculty of Psychology, University of Akureyri, Nordurslóð 2, 600, Akureyri, Iceland; b Department of Neurology, Christian Doppler Medical Centre and Centre for Cognitive Neuroscience, Paracelsus Medical University, Salzburg, Austria; c Laboratoire EMC, Mémoire, Émotion et Action, Université Lumiére Lyon 2, Lyon, France

**Keywords:** Virtual reality, Epilepsy monitoring unit, Episodic memory, Delayed recall

## Abstract

In the epilepsy monitoring unit of the Department of Neurology at the University Clinic of Salzburg 20 adult patients were recruited to participate in a validation of 6 parallel versions of the virtual reality test for episodic memory. Patients were tested up to 7 times, i.e. twice a day, in the morning and evening, beginning on Monday evening. Each session consisted of learning a new town and immediate recall for this town. All sessions but the first one included also delayed recall of the previously learned town and a recognition test. Recall included the sub-scales what, details, when, egocentric where and allocentric where. Recognition memory was tested by presenting the patients 30 sentences of which 15 were true and 15 were false.

While not all patients completed the full testing schedule, at immediate recall for 9 patients a full data set (7 sessions) is available. All patients were free of antiepileptic medication (N = 19) or medication was kept constant across the week (N = 1).

This data can be used to demonstrate the feasibility to use the virtual reality test in the epilepsy monitoring unit e.g. to monitor effects of seizures or medication on episodic memory.

**Table:** Specifications Table

Subject	Neuropsychology and Physiological Psychology
Specific subject area	Testing of episodic memory in the epilepsy monitoring unit with virtual reality techniques and parallel versions of a test.
Type of data	Table (CSV) Figure
How data were acquired	Data was acquired bed-site. Exposure to the virtual environment was done with a Lenovo Laptop (17inch), which was also used to present the recognition task. Recognition was tested with the software Presentation (Neurobehavioral systems). Recall was tested in a structured interview according to the attached table. In the learning part of the virtual reality test, participants navigated through a virtual town. The task and its six versions are conceptually an update and an extension to six parallel versions of the virtual reality test as presented previously [1] and are freely available on Mendeley [2]. For creating six parallel towns, we used UNITY (Unity Technologies ApS, unity3d.com), including several packages (asset store products) in order to fill the towns with details, such as the supply low poly dudes, character pack one and two, contemporary city, super market, European city buildings, and a power station. We sorted the available details in order to fill 10 scenes per town.
Data format	Raw Analyzed
Parameters for data collection	The participants were tested on seven occasions for four consecutive days, usually from Monday evening until Thursday evening. Test sessions took place at 6–8pm and 7–9am. In the first session, the learning part was conducted, followed by immediate recall. The next session started with delayed recall and recognition, and then learning and immediate recall of a second version was presented. The order of the six parallel versions was the same for all participants.
Description of data collection	In the learning part participants were instructed to move forward, not to stop, and not to walk too fast. The time spent in town was recorded automatically by UNITY for later analysis. We informed participants a priori about the questions we would ask in the recall part. Recall requires the participants to spell out remembered details, which were noted on a structured grid of responses. For each of the dimensions WHAT, DETAILS, WHEN, ALLOCENTRIC WHERE, and EGOCENTRIC WHERE, participants were asked specific questions, and the number of correctly reported items were evaluated, rated, and counted from the structured grids of responses.
Data source location	Institution: Department of Neurology, Christian Doppler Medical Centre and Centre for Cognitive Neuroscience, Paracelsus Medical University Salzburg City/Town/Region: Salzburg Country: Austria
Data accessibility	Data is with the article. Additionally, the virtual town task is available in the following repository: Repository name: Mendeley Data Data identification number: https://doi.org/10.17632/4nt4t3zs8f.1 Direct URL to data: https://data.mendeley.com/datasets/4nt4t3zs8f/1

**Table:** 

**Value of the Data** • These data demonstrate a range of values that can be obtained by using the virtual reality test, and its feasibility in the epilepsy monitoring unit. • Researchers considering using the virtual reality task can access the range of remembered items in Fig. 1, Fig. 2, Fig. 3, Fig. 4, Fig. 5, Fig. 6, Fig. 7, Fig. 8, Fig. 9, Fig. 10, Fig. 11 or use the raw data in order to generate more information on the distribution of values. Knowing the distribution can be useful when planning future studies, e.g. in order to estimate an effect size for power calculation. • The present data, alongside with the towns, can be used to improve the virtual reality test versions in order to make them even more parallel.

## 1 Data description

Out of the N = 20 patients without a change in medication, N = 9 had a complete dataset (i.e. participated in all 6 towns). All but one of these patients took no drugs at all, one had a constant antiepileptic drug load of dd = 2 (patient with ID = 18).

File metadata.csv in the supplementary data holds the information concerning age and gender of the participants. Age is given in years for age at testing, and gender is given in numbers (0 for female and 1 for male).

File data.csv in the supplementary data holds the respective raw data for the subscales of the virtual reality test. Columns indicate patient number, session number, WHAT immediate, DETAILS immediate, WHEN immediate, ALLOCENTRIC WHERE immediate, and EGOCENTRIC WHERE immediate, WHAT delayed, DETAILS delayed, WHEN delayed, ALLOCENTRIC WHERE delayed, EGOCENTRIC WHERE delayed, and RECOGNITION which exists only for the delayed recall, and time, which indicates the time spent in each town.

Fig. 1, Fig. 2, Fig. 3, Fig. 4, Fig. 5, Fig. 6, Fig. 7, Fig. 8, Fig. 9, Fig. 10, Fig. 11 show the boxplots for performance for all subscales, immediate and delayed recall, across the 6 versions:

**Fig. 1:**
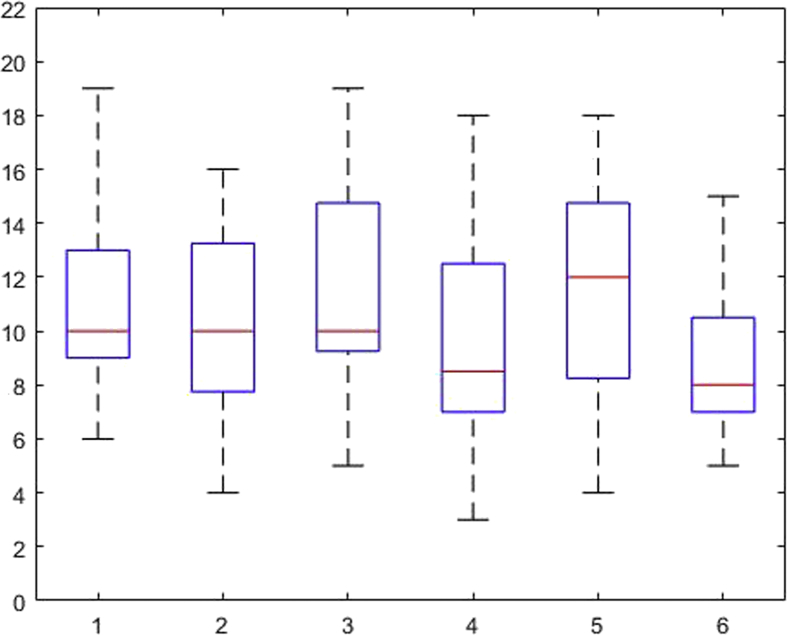
What immediate.

**Fig. 2:**
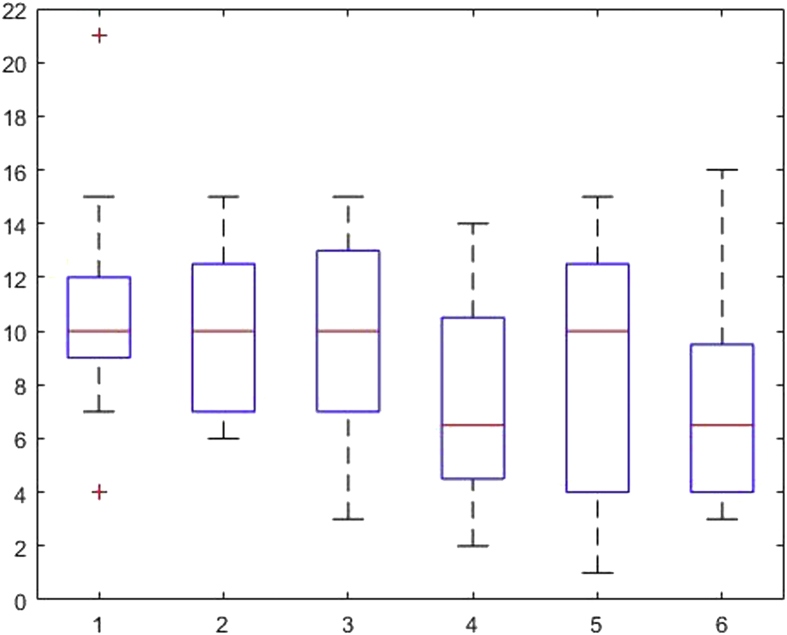
What delayed.

**Fig. 3:**
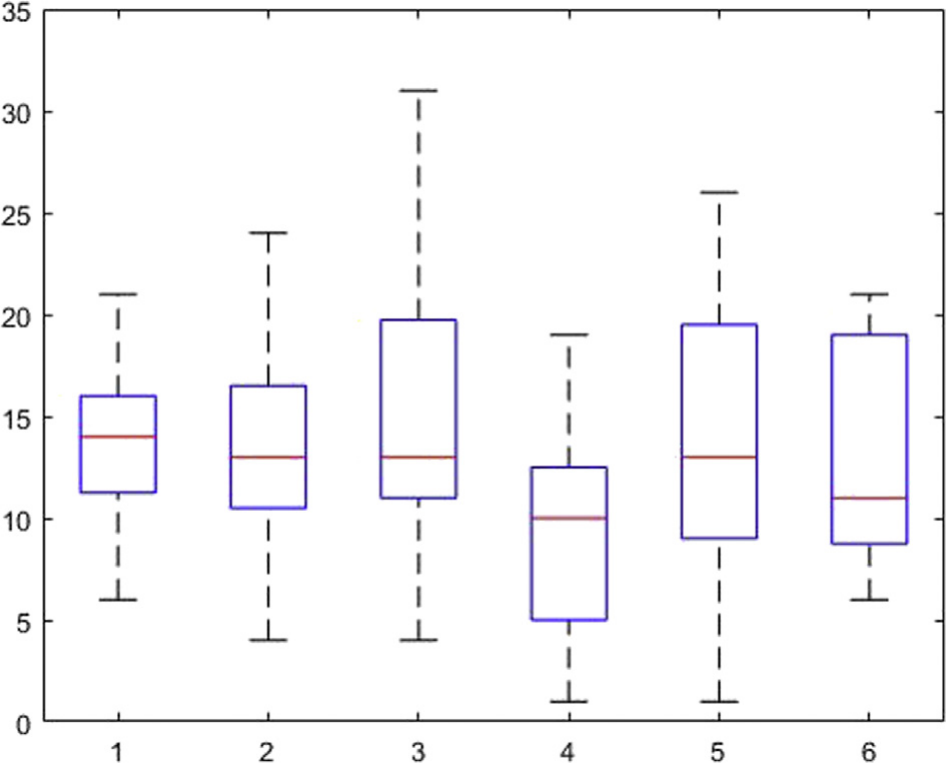
Details immediate.

**Fig. 4:**
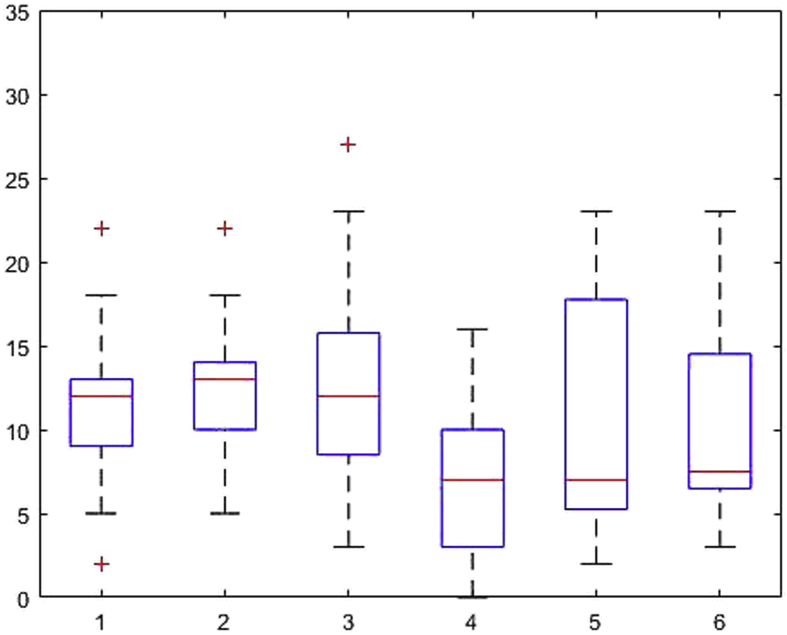
Details delayed.

**Fig. 5:**
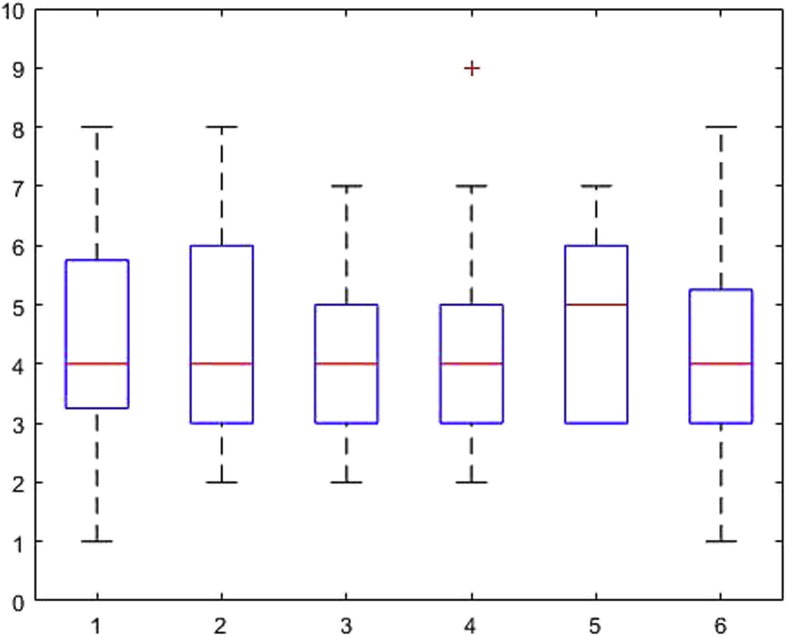
When immediate.

**Fig. 6:**
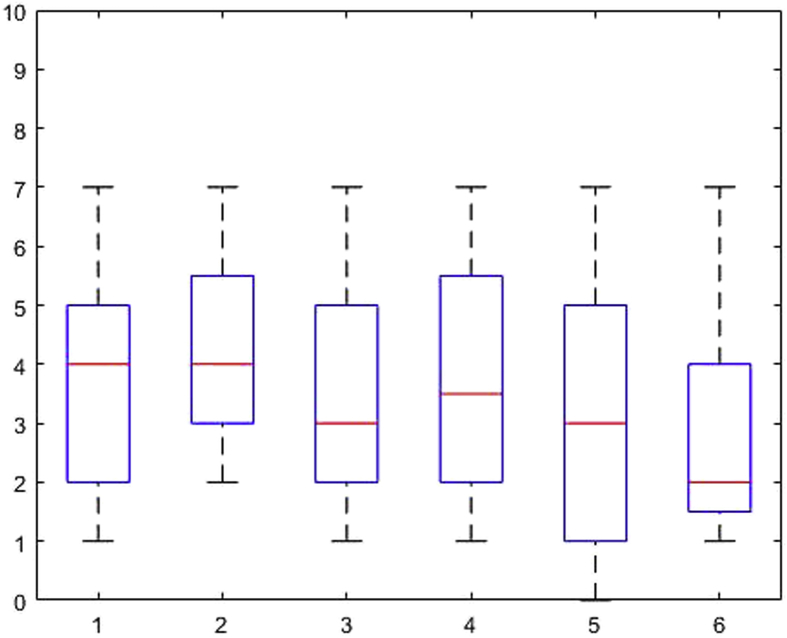
When delayed.

**Fig. 7:**
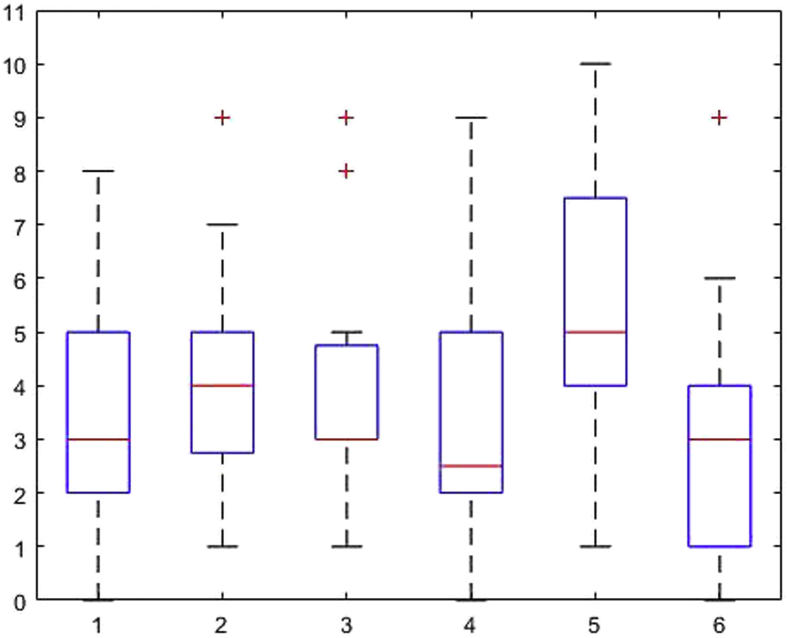
Allocentric where immediate.

**Fig. 8:**
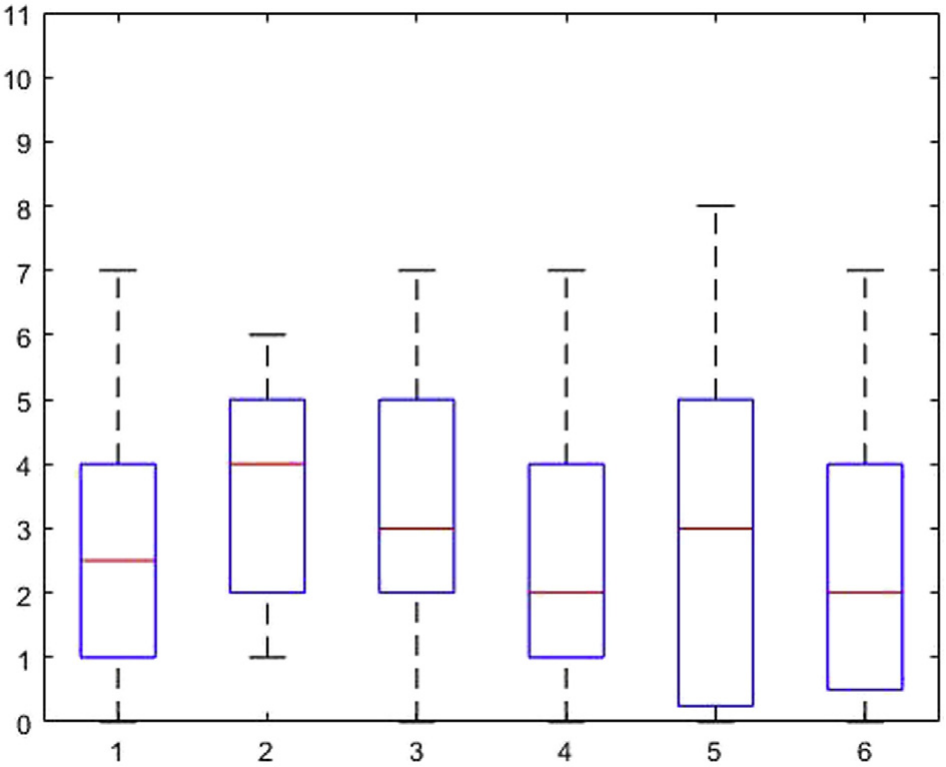
Allocentric where delayed.

**Fig. 9:**
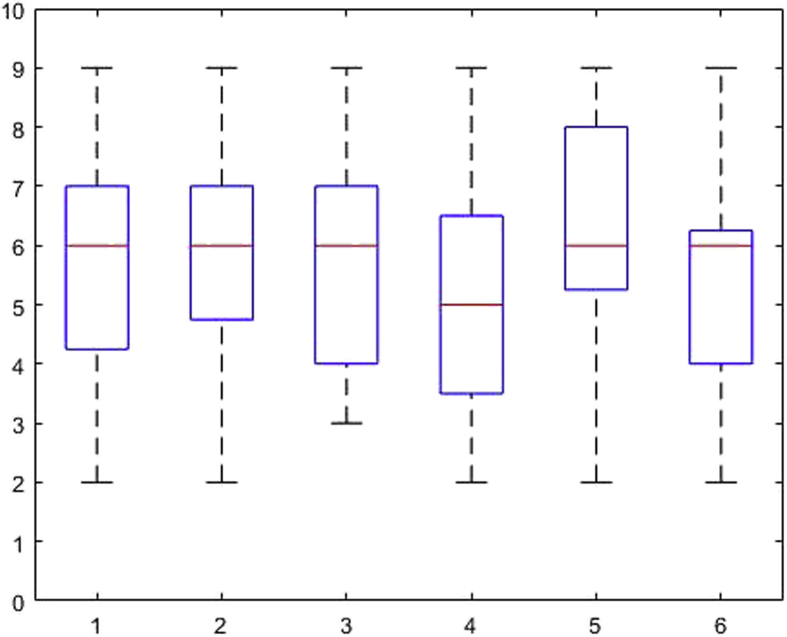
Egocentric where immediate.

**Fig. 10:**
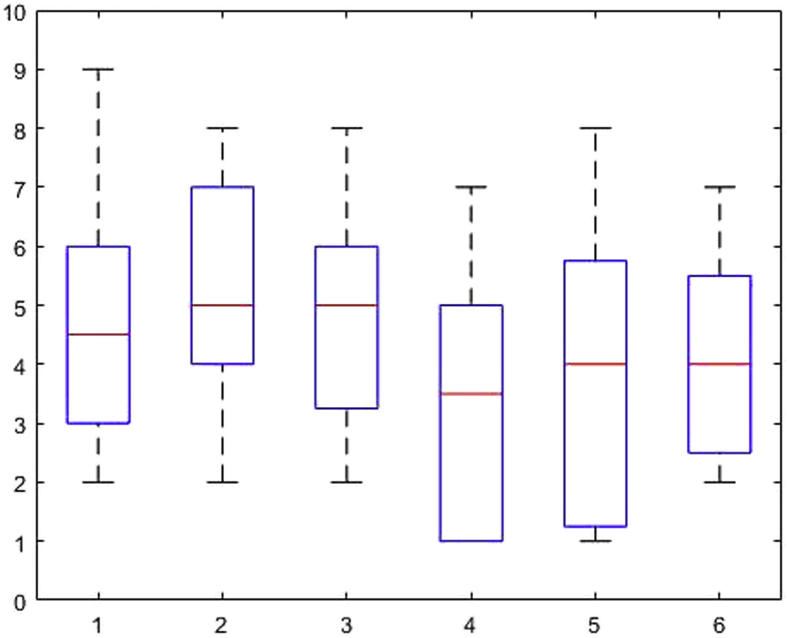
Egocentric where delayed.

**Fig. 11:**
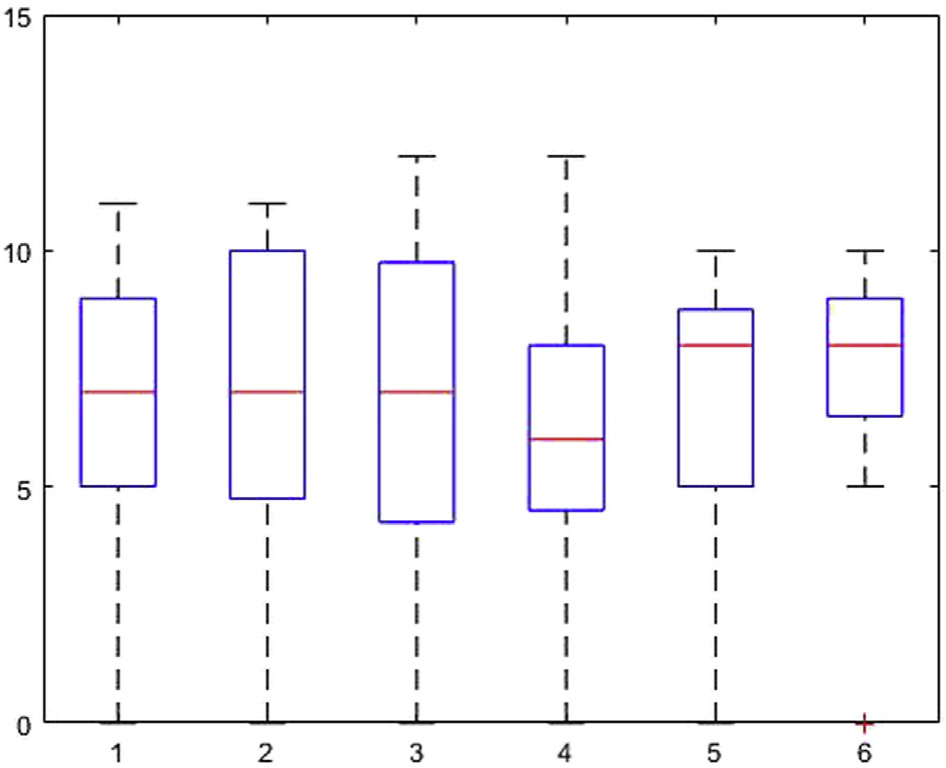
Recognition delayed.

The x-axis indicates the sessions, i.e. the 6 task versions, the y-axis the number of correctly remembered items. On each box, the central mark indicates the median, and the bottom and top edges of the box indicate the 25th and 75th percentiles, respectively. The whiskers extend to the most extreme data points not considered outliers, and the outliers are plotted individually using the '+' symbol.

## 2 Experimental design, materials, and methods

### 2.1 Setting

This data collection was conducted in the routine care of the epilepsy monitoring unit of the Department of Neurology, Christian Doppler Medical Centre, Salzburg, Austria. Admitted patients undergo the usual diagnostic evaluations consisting of long-term video-EEG monitoring. Recordings are performed over a maximum period of five days (Monday to Friday). In order to promote a timely occurrence of seizures during the monitoring period, it is common practice to taper the dosage of antiepileptic drugs and expose patients to sleep deprivation. Informed consent for serious adverse events was completed routinely upon admission.

### 2.2 Ethical aspects

The data collection was designed and conducted in accordance with the World Medical Association Declaration of Helsinki and Good Clinical Practice Guidelines. Prior ethical approval was obtained from the ethical committee Salzburg (415-E/1755/24–2018). Written informed consent was obtained from all participants. The data is presented in anonymous form and it is not possible to link the data to individual patients.

### 2.3 Recruitment of patients

A consecutive sample of 106 patients was enrolled for data collection. All patients who were 18 years and older, able to give written informed consent, and who were admitted to the epilepsy monitoring unit between February 2, 2016 and June 11, 2018 were considered for recruitment. Patients are admitted to the epilepsy monitoring unit in order to classify the epilepsy syndrome, for differential diagnosis of suspicious events, for the assessment of seizure frequency, for optimization of medication, or for presurgical evaluation. Each week, at most four patients were admitted to the epilepsy monitoring unit and at most one patient was enrolled. We strived to recruit patients with already ascertained diagnosis of epilepsy, or who fitted well into the control group because a diagnosis of epilepsy was unlikely. Based on these criteria we approached the best suited patient first. The present data in brief article presents data of the control group.

Participants which were released from the epilepsy monitoring unit with the diagnosis “no epilepsy” were included in the control group. These patients were admitted to the epilepsy monitoring unit due to a single event of unclear nature. Participants in the control group did not have seizures or interictal epileptiform events during their stay in the monitoring.

Neurological examination included in most cases also structural magnetic resonance imaging and in some patients with intractable epilepsy also single-photon emission computed tomography, positron emission tomography, and neuropsychological examination.

### 2.4 Procedure

The participants were tested at bedside with a Lenovo Laptop (17inch) on seven occasions for four consecutive days, usually from Monday evening until Thursday evening. Test sessions took place in the evening between 6 and 8pm, and in the morning between 7 and 9am. The test sessions included a questionnaire about subjective feelings of stress and tiredness, a fingertapping task, a verbal memory task, and the virtual reality test, where only the latter is evaluated and presented here. In the first session, for all three tasks the learning part was conducted. The verbal memory task and the virtual reality task were followed by immediate recall. The next session started with delayed recall for all three tasks, and then a second version of all three tasks was presented for learning, as well as immediate recall for the verbal memory task and the virtual reality task. The procedure and the order of the six parallel versions was the same for all participants.

### 2.5 Virtual reality test

In the learning part of the virtual reality test, participants navigated through a virtual town. The task and its six versions are conceptually an update and an extension to six parallel versions of the virtual reality test as presented previously [1] and are freely available on Mendeley [2].

**Fig. 12:**
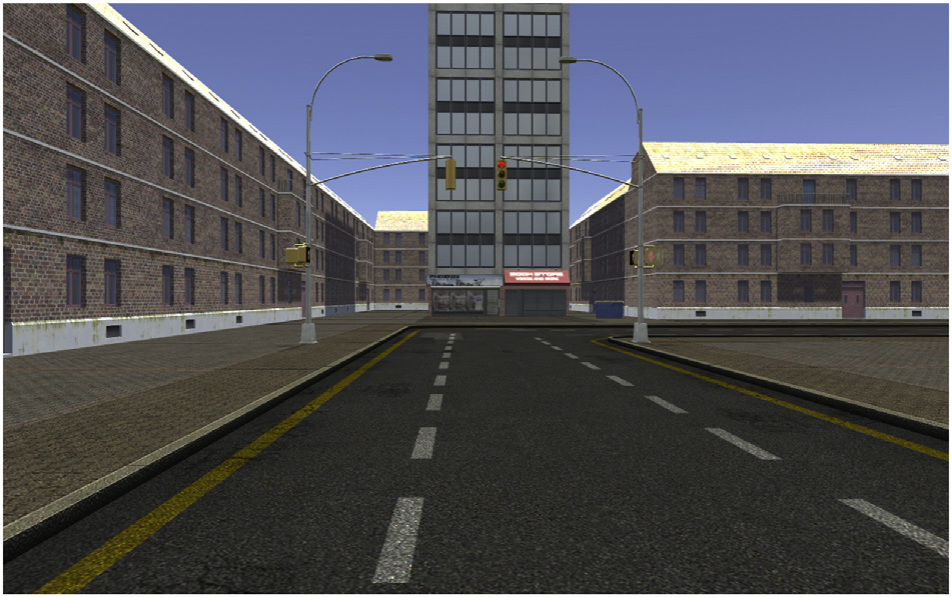
Exemplar view from virtual town version one.

For creating six parallel towns, we used UNITY (Unity Technologies ApS, Unity3d.com), including several packages (asset store products) in order to fill the towns with details, such as the supply low poly dudes, character pack one and two, contemporary city, super market, European city buildings, and a power station. We sorted the available details in order to fill 10 scenes per town (9 scenes in prior work [1]). In all towns, participants could follow only the main street, there were no options to choose a turn or to get lost. Each scene was at a turn of the street and included at least two elements. An exemplar view from town 1 is shown in Fig. 12. In the second scene participants could remember a kiosk (element 1), and right to it (allocentric information) two tables with benches (element 2). Behind the benches (allocentric information) there is a tree (element 3). The kiosk is a small house (detail), the benches are each two for each of the two tables (details). After this scene, participants turned left (egocentric information).

**Fig. 13:**
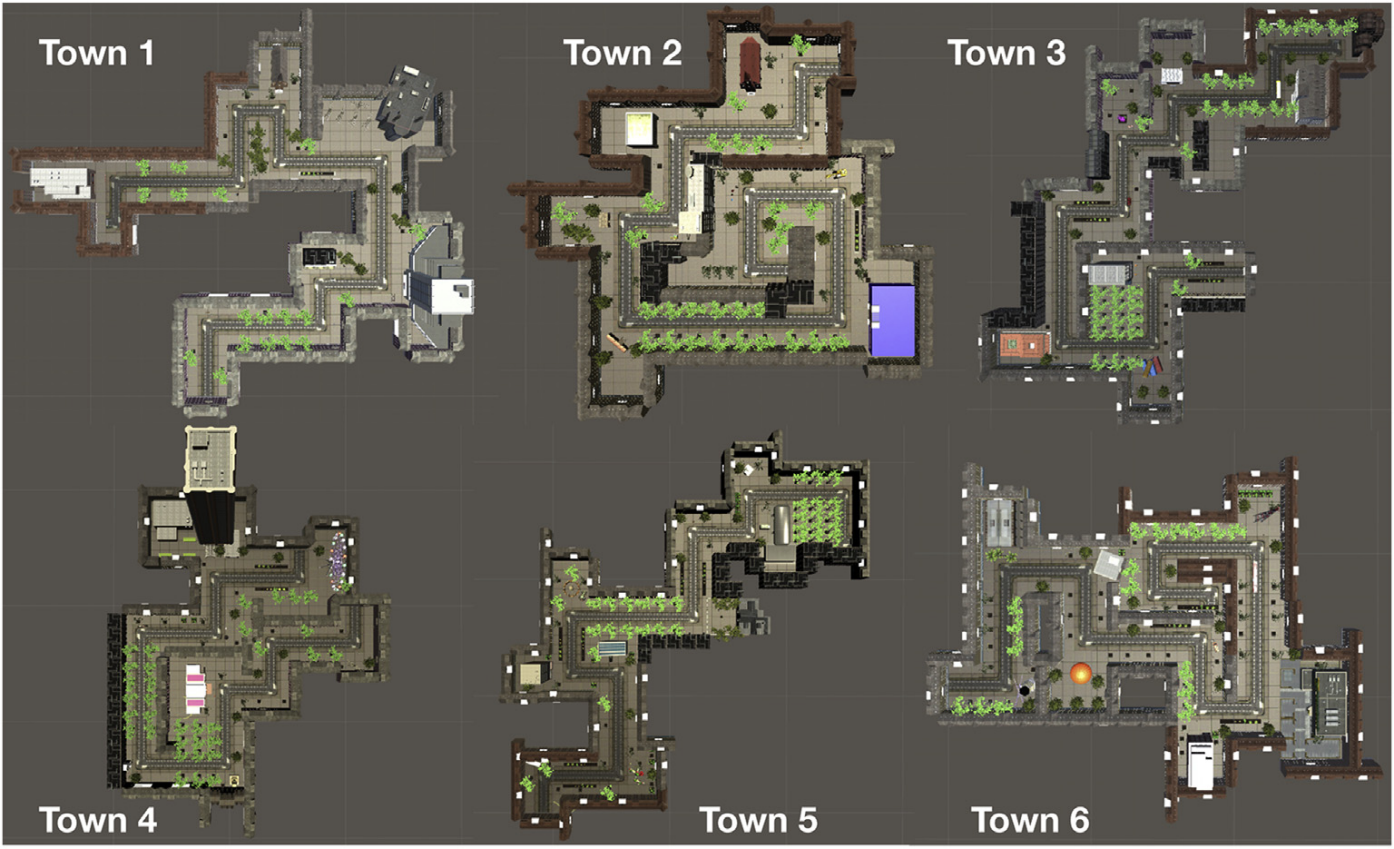
Outline of the six parallel versions of the test, i.e. the six towns.

The total number of elements was balanced across the 10 scenes and six towns. The outline of the six towns is shown in Fig. 13. The environment was explored in pedestrian mode, participants used the cursor control keys to navigate.

We also created an empty town of rectangular shape which allowed to go in circles. This town was used on the first day of examination prior to beginning with the learning part of the first town, in order to familiarize participants with the environment and navigation. In order not to confuse participants with details, there were no details in this training town. Thus, it consisted only of the street as well as houses to the left and right of the street. The participants moved in the training town until they felt comfortable with the virtual reality environment and navigation, at least for one loop through the town.

In the learning part of the virtual reality task, participants were instructed to move forward, not to stop, and not to walk too fast. The time spent in town was recorded automatically by UNITY for later analysis. We informed participants a priori about the questions we would ask in the recall part.

The recall-part of this test was performed immediately after exploring the town and again about 12 hours later. Recall requires the participants to spell out remembered details, which were noted on a structured grid of responses. The participants were asked:

**WHAT**: *What did you see in the virtual town?* Patients reported elements they remembered, such as, e.g. a kiosk, benches, a hospital, a gas station, a traffic light, etc.

**DETAILS**: *Can you describe the* < *element> in more detail?* For each element that was noted, patients were asked which details they remembered that describe the element better.

**WHEN**: *When did you see the* < *element* > *, at the beginning, in the middle, or at the end of the town?* For each element they reported, patients were asked when the element had occurred. They were encouraged to judge whether it occurred in the beginning, middle or towards the end of the walk through the town.

**ALLOCENTRIC WHERE**: *Was there anything close to the* < *element> and if so, was that left or right, before or behind the* < *element* > ? Patients were asked about the arrangement of the elements to each other in case they occurred together in a scene.

**EGOCENTRIC WHERE**: *After the* < *element* > *, did you turn left or right?* Patients were asked to remember if they turned left or right after the element.

For each of the dimensions WHAT, DETAILS, WHEN, ALLOCENTRIC WHERE, and EGOCENTRIC WHERE, the number of correctly reported items were evaluated, rated, and counted from the structured grids of responses. At the end of data collection, the authors YH and CH evaluated all responses and preliminary evaluations, in order to agree on one evaluation strategy that allowed a consistent appraisal across all patient's responses. For example, number of details turned out to be inconsistent across several raters such that it was necessary to define a set of valid details. Furthermore, for WHEN the description of a scene being in the beginning, middle, or end was arbitrary from the outset. In an attempt to improve the accuracy of the WHEN scale we standardized the evaluation: For “beginning” we matched the first 3 scenes, for “middle” the middle 4 scenes, and for “end” the last 3 scenes. The consensus list of valid items was applied to all patient's responses by one rater (co-author CH), in order to avoid inter-rater bias.
